# Increased rate of day surgery use for inguinal and femoral hernia repair in a decade of hospital admissions in the Veneto Region (north-east Italy): a record linkage study

**DOI:** 10.1186/1472-6963-13-349

**Published:** 2013-09-12

**Authors:** Mario Saia, Domenico Mantoan, Alessandra Buja, Chiara Bertoncello, Tatjana Baldovin, Chiara Zanardo, Giampietro Callegaro, Vincenzo Baldo

**Affiliations:** 1 Veneto Region Health Directorate, Sanità Regione Veneto, Palazzo Molin San Polo, 2513 - 30125 Venezia, VE, Italy; 2 Department of Molecular Medicine, Laboratory of Public Health and Population Studies, University of Padova, Padova, Via Loredan 18 35127 Italy; 3, Local Health Unit No. 8, Via dei Carpani 16/Z, 31033 Castelfranco Veneto, Veneto Region, Italy

**Keywords:** Epidemiology, Groin hernia, Inguinal hernia, Femoral hernia, Day surgery, Trend analysis

## Abstract

**Background:**

Worldwide, there has been a marked increase in the number of inguinal and femoral hernia repairs performed as day surgery procedures. This study aimed to outline the epidemiology of the procedures for repairing unilateral inguinal and femoral hernia in the Veneto Region, and to analyze the time trends and organizational appropriateness of these procedures.

**Methods:**

Drawing from the anonymous computerized database of hospital discharge records for the Veneto Region, we identified all unilateral groin hernia repair procedures completed in Veneto residents between 2000 and 2009 at both public and accredited private hospitals.

**Results:**

A total 141,329 hernias were repaired in the Veneto Region during the decade considered, with an annual rate of 291.2 per 100,000 population for inguinal hernia (IH) repairs and 11.2 per 100,000 population for femoral hernia (FH) repairs. Day surgery was used more for inguinal than for femoral hernia repairs, accounting for 76% and 43% (p< 0.05), respectively, of all hernia repair procedures completed during the period. The % of other than surgery hospital ordinary admissions (day surgery or ambulatory surgery) during the decade considered rose from 61.7% to 86.7% for IH and from 33.0% to 61.8% for FH.

**Conclusions:**

In the last decade, the Veneto Region has reduced the rate of ordinary hospital admissions for groin hernia repair with a view to improving the efficiency of the hospital network.

## Background

With advances in diagnostic and therapeutic technologies, and surgical and anesthetic techniques, there has been a reduction in the number and duration of conventional hospitalizations and an increasing use of day surgery and ambulatory surgery procedures. This is due to the application of organizational models that pursue the efficient management of resources among their main objectives. One way to do so is to diversify the flow of patients for surgery based on the concept of appropriateness in terms not only of clinical aspects, but also of organizational issues, with a view to making the best possible use of the available health services [1].

Guidelines drawn up by the Royal College of Surgeons in the UK argue that day surgery is the ideal treatment approach from the point of view of patient care, efficiency, and patient satisfaction for numerous operations, and the best choice for 50% of patients having to undergo surgery [2].

Day surgery and outpatient or ambulatory solutions can preserve or even increase the level of service quality in terms of outcomes, while reducing any negative consequences relating to prolonged hospital stays. They can also lead to shorter waiting lists, and help to rationalize the costs of surgical treatments. In the case of inguinal and femoral hernia repair, the European Hernia Society has published a grade B (“weakly positive”) recommendation that all patients be considered potentially eligible for day surgery [3].

The advantages of inguinal hernia (IH) repair as a day surgery procedure, i.e. quicker mobilization, patient satisfaction and cost reduction, had been described in the literature already in 1955 [4]. Observational studies have quantified the cost savings achievable with this approach, showing that surgery for IH repair in patients staying in hospital costs 56% more than day surgery [5,6].

Another European study showed an €800 saving for every hernia repaired at a day surgery (DS) clinic instead of having patients admitted for conventional hospital care [7]. In terms of the efficiency of this organizational model, several randomized studies compared the DS procedure with traditional hospitalization years ago [8,9], and a more recent randomized trial also considered the value of IH patients’ preferences for one or other procedure [10]. These studies all showed that, as well as being economically advantageous, surgery in outpatients is just as safe and effective as inpatient surgery.

On a worldwide basis, there has been a clear increase in the proportion of IH repair procedures performed at DS clinics [11,12]. There is a marked diversity in this proportion between different countries, however, which is not only attributable to patients’ and surgeons’ acceptance of the DS option, but also depends largely on how the health services are financed. Between the years 2000 and 2004, 35% of IH repairs in the Netherlands and 33% in Spain were DS procedures. In the Swedish National Registry, 75% of IH repairs were done at DS clinics. An OECD publication in 2003 confirmed an increasing recourse to DS between 1996 and 2000 for several procedures, including inguinal and femoral hernia repair, for which the proportion of cases treated as outpatients rose from 45.3% to 64.2% [13]. In Italy too, one of the principal goals of the health system managers is to assess and monitor the organizational appropriateness of transferring some surgical procedures from ordinary hospitalization to other health care solutions. The promotion of day surgery varies considerably in different parts of the country, however, partly due to some hospital administrations tending to resist the need to adapt their role in a rapidly changing and evolving situation [14].

The aim of this study was to describe the epidemiology of unilateral IH and femoral hernia (FH) repair in the Veneto Region (North-Eastern Italy) and to analyze how the health care services for these surgical procedures have changed over time.

## Methods

### The regulatory environment

In accordance with the provisions of the Presidential Decree of 20 October 1992 [15], the Veneto Regional Council took steps in 1996 to regulate the development of day surgery, having established the rules for admitting patients for ambulatory care services [16,17]. Already by 1998, a number of changes had been made [18], and surgery for the bilateral and unilateral repair of both IH and FH had been listed among the procedures that could be handled in a DS setting.

The repair of unilateral IH and FH in outpatients was just one of a number of actions taken by the health services in 2007 to ensure more appropriate healthcare dispensing based on criteria of clinical appropriateness, cost effectiveness and the efficient use of resources [19]. These provisions allow for outpatient hernia repair in patients meeting the requirements of the DRG 162 “Inguinal and Femoral Hernia, age >17, no complications”, and specific codes referring to the specialist branch of “general surgery” were included in the NHS Regional price list for ambulatory services. Generally speaking, “ambulatory surgery” is not associated in Italy with a formal hospital admission, it is considered an outpatient service, whereas “day surgery” is regarded as coinciding with a hospital admission lasting one day, with patients being discharged on the same calendar day.

### Analytical methods

Using the anonymous computerized database of the Veneto Region’s hospital discharge records as a source (the database is not publicly available), we identified all unilateral IH and FH repairs performed in the decade between 2000 and 2009 in Veneto residents at both public and accredited private facilities.

At the same time, we conducted a search in the regional computerized archives of outpatient data and identified all the same hernia repairs completed as ambulatory procedures in 2008–2009.

Steps were taken to analyze the care delivery system for hernia repairs based on the patients’ personal characteristics, and to calculate the annual intervention rate (AIR) per 100,000 population, assessing the progress made in the decade considered.

To see whether the amount of time spent in hospital for scheduled surgical procedures has changed over the years for the various types of care-providing service, we adopted a proxy indicator of healthcare service efficiency called the “mean days of admission indicator”, meaning the actual number of days spent in hospital or at ambulatory services, including:

– days of ordinary hospitalization, disregarding urgent admissions (that, by definition, could not be transferred to outpatient or ambulatory services);

– days spent in day surgery clinics, not counting the days when patients go for preoperative and postoperative visits (although this entails a consumption of resources);

– days of outpatient ambulatory attendance.

A suitable descriptive analysis was performed to calculate the distribution of absolute and relative frequencies for categorical variables and the average for quantitative variables. The t-test or F-test was used to check for any differences in mean hospital stay between the groups, and any evidence of a time trend was measured as the average annual percent change (using the Joinpoint Regression Program).

### Ethical issues

The study was carried out on data routinely collected by the health services on anonymized records without any chance of individuals being identified. The data analysis was performed on aggregated data. The data in the Local Health Authority registries are recorded with the patient’s consent and can be used as aggregated data for scientific studies without further authorization (*Garante per la protezione dei dati personali,* Resolution of 1 March 2012, n. 85). The study complies with the Declaration of Helsinki and with the Italian Decree n. 196/2003 on the protection of personal data.

## Results

During the decade from 2000 to 2009, 143,910 Veneto residents underwent IH or FH repair. After excluding 2,581 operations (1.8%) performed outside the region, the total number of surgical procedures for hernia repair performed in the Veneto was 141,329 (136,075 for IH and 5,254 for FH); 132,569 of these cases involved unilateral hernias.

The AIR (Table 1) was calculated on the total number of unilateral and bilateral procedures performed within the region: the overall AIR for IH repair amounted to 291.2 procedures per 100,000 population. Patients were predominantly male (89% of the procedures) and the age groups with the highest AIR was 65–84 years old for males. The AIR for FH repair amounted to 11.2 per 100,000 population, and 69% of the procedures were performed in female patients. Table 1 also shows the distribution by gender and age group of the procedures performed in both inpatients and outpatients, showing that IH repair was involved in 96% of all the procedures.

**Table 1:** Annual intervention rate (AIR) for inguinal and femoral hernia repair by sex and age group

**Inguinal hernia**	**Women**				**Men**				**Total**	
	**Unilat**	**Bilat**	**Total**		**Unilat**	**Bilat**	**Total**			
	**n**	**n**	**n**	**AIR**	**n**	**n**	**n**	**AIR**	**n**	**AIR**
0 y	92	232	324	148.8	1190	154	1344	584.9	1668	372.7
01-05 y	953	375	1328	124.6	3392	145	3537	313.4	4865	221.7
06-14 y	513	80	593	32.3	1600	39	1639	84.4	2232	59.1
15-24 y	262	7	269	12.2	2090	69	2159	92.9	2428	53.5
25-44 y	2063	74	2137	29.5	18572	1113	19685	257.5	21822	146.6
45-64 y	3612	129	3741	61.5	44316	3274	47590	789.4	51331	423.9
65-74 y	2754	155	2909	113.6	26495	1714	28209	1318.5	31118	662.1
75-84 y	2455	101	2556	133.2	14264	785	15049	1324.4	17605	576.4
> 85 y	646	29	675	88.5	2215	116	2331	865.8	3006	291.3
Total	13350	1182	14532	60.8	114134	7409	121543	532.1	136075	291.2
**Femoral hernia**	**Women**				**Men**				**Total**	
	**Unilat**	**Bilat**	**Total**		**Unilat**	**Bilat**	**Total**			
	**n**	**n**	**n**	**AIR**	**n**	**n**	**n**	**AIR**	**n**	**AIR**
0 y	0	1	1	0.5	1	0	1	0.4	2	0.4
01-05 y	11	0	11	1.0	6	3	9	0.8	20	0.9
06-14 y	13	0	13	0.7	10	0	10	0.5	23	0.6
15-24 y	30	0	30	1.4	11	2	13	0.6	43	0.9
25-44 y	498	14	512	7.1	161	7	168	2.2	680	4.6
45-64 y	976	33	1009	16.6	508	20	528	8.8	1537	12.7
65-74 y	741	13	754	29.4	409	24	433	20.2	1187	25.3
75-84 y	839	33	872	45.5	351	12	363	31.9	1235	40.4
> 85 y	432	5	437	57.3	88	2	90	33.4	527	51.1
Total	3540	99	3639	15.2	1545	70	1615	7.1	5254	11.2

The bilateral procedures apparently included more frequency of IH surgery in females (8.8% vs. 6.5% in males; p^χ2^ <0.05), and of FH surgery in males (4.5% vs. 2.8% in female; p^χ2^ <0.05). The 8,760 bilateral repair procedures involved more frequency of IH than FH (6.3% vs. 3.2%, respectively, of the whole sample considered; p^χ2^ <0.05). Being ineligible for day surgery, all cases of bilateral hernia were excluded from the subsequent analyses.

Table 2 shows the distribution by age group, type of healthcare service, and usage of synthetic grafts for the 124,789 unilateral repairs performed in inpatients, which involved 120,112 IH (90.6%) and 4,677 FH (9.4%). It is clear from the table that day surgery was used more for repairing IH than for FH during the decade considered (76% and 43%, respectively; p^χ2^ <0.05).

**Table 2:** Unilateral surgery for hernia repair by type of admission

**Inguinal hernia**	**Total**	**With mesh**						**Without mesh**					
		***Admission >1 day***		***Day-surgery***		**Total**	**Day-surgery**	***Admission >1 day***		***Day-surgery***		**Total**	**Day-surgery**
	**n**	**n**	**Mean LOS**	**n**	**Mean accesses**	**n**	**%**	**n**	**Mean LOS**	**n**	**Mean accesses**	**n**	**%**
0 y	**1282**	7	2.4	2	2.0	9	22	702	3.4	571	2.2	1273	45
01-05 y	**4345**	19	2.6	15	2.3	34	44	1424	1.9	2887	2.5	4311	67
06-14 y	**2108**	19	1.9	76	1.8	95	80	514	2.0	1499	2.4	2013	75
15-24 y	**2216**	316	2.0	1754	1.8	2070	85	41	2.4	105	2.0	146	72
25-44 y	**19289**	2667	2.1	15952	1.8	18619	86	185	2.9	485	1.8	670	72
45-64 y	**44727**	7683	2.4	35686	1.8	43369	82	491	3.3	867	2.1	1358	64
65-74 y	**27396**	6554	3.0	19906	1.8	26460	75	453	3.8	483	1.9	936	52
75-84 y	**15972**	5522	3.5	9790	1.9	15312	64	414	5.9	246	1.7	660	37
≥ 85 y	**2777**	1477	4.5	1166	1.8	2643	44	111	7.3	23	1.3	134	17
***Total***	***120112***	***24264***	***2.9***	***84347***	***1.8***	***108611***	***78***	***4335***	***3.1***	***7166***	***2.3***	***11501***	***62***
**Femoral hernia**	**Total**	**With mesh**						**Without mesh**					
		***Admission >1 day***		***Day-surgery***		**Total**	**% Day-surgery**	***Admission >1 day***		***Day-surgery***		**Total**	**% Day-surgery**
	**n**	**n**	**Mean LOS**	**n**	**Mean accesses**	**n**		**n**	**Mean LOS**	**n**	**Mean accesses**	**n**	
0 y	**1**							1	1.0			1	0
01-05 y	**17**							6	1.7	11	2.5	17	65
06-14 y	**22**			3	1.0	3	100	9	2.1	10	1.6	19	53
15-24 y	**37**	10	2.6	16	1.6	26	62	3	1.7	8	1.1	11	73
25-44 y	**597**	144	2.1	331	1.8	475	70	35	2.5	87	1.6	122	71
45-64 y	**1290**	460	2.9	608	1.8	1068	57	98	4.1	124	1.6	222	56
65-74 y	**1040**	466	3.8	373	1.9	839	44	126	4.3	75	1.6	201	37
75-84 y	**1156**	621	4.9	282	1.8	903	31	200	7.3	53	1.6	253	21
≥ 85 y	**517**	339	6.2	33	1.8	372	9	140	8.5	5	1.4	145	3
***Total***	***4677***	***2040***	***4.2***	***1646***	***1.8***	***3686***	***45***	***618***	***6.0***	***373***	***1.6***	***991***	***38***

LOS = length of stay.

For both types of hernia, hospitalization was longer for meshless procedures (p_t-student_ < 0.05), and IH patients’ mean hospital stay (disregarding the particular cohort of patients under five year old) increased with age (p < 0.05), irrespective of the surgical technique used.

Table 3 summarizes the data for the decade considered in our Region, showing the procedures performed in both inpatients and outpatients. There was only a significant drop in the AIR for IH repair from 2002 (p_forAPC_ <0.05), while the AIR for FH remained substantially unchanged. The table also shows the urgent procedures in inpatients, which involved FH more often than IH (p^χ2^ <0.05), with 67% and 18% of all the inpatient procedures, respectively. The length of hospital stay (LOS) amounted to 3 ± 1.12 days for IH and 4.6 ± 1.9 days for FH, and did not change significantly during the period analyzed. For urgent admissions, the LOS remained much the same during the period considered, and stood at 5.3 ± 2.4 days for IH and 5.1 ± 2.14 days for FH (data not shown).

**Table 3:** Distribution of 2000–2009 hernia repairs

**Inguinal hernia**	**Year**									
	**2000**	**2001**	**2002**	**2003**	**2004**	**2005**	**2006**	**2007**	**2008**	**2009**
***Total number***	11652	12790	12800	12917	13023	13027	12597	13255	12612	12811
rate x 100,000/year	258.3	281.7	282.6	282.2	280.5	277.2	265,9	277,7	261,0	262,2
***Ordinary admission (n.)***	4462	4276	3902	3031	2671	2497	2386	2088	1589	1697
Day of stay	15097	13000	10616	8159	7569	6953	6659	5810	5269	5251
Mean Length of stay	3.4	3.0	2.7	2.7	2.8	2.8	2.8	2.8	3.3	3.1
% Ordinary admission	38.3%	33.4%	30.5%	23.5%	20.5%	19.2%	18.9%	15.8%	12.6%	13.2%
% Urgent ordinary admission	15%	14%	14%	17%	18%	20%	20%	22%	30%	28%
***Day surgery (n.)***	7190	8514	8898	9886	10352	10530	10211	11167	8394	6371
n. visit	13749	16324	17101	18150	19056	19832	19668	20433	14566	11237
% Day surgery	61.7%	66.6%	69.5%	76.5%	79.5%	80.8%	81.1%	84.2%	66.6%	49.7%
***Ambulatory (n)***									2629	4743
% ambulatory									20.8%	37.0%
% Not ordinary admission	61.7%	66.6%	69.5%	76.5%	79.5%	80.8%	81.1%	84.2%	87.4%	86.7%
Mean admission days indicator	1.7	1.5	1.4	1.3	1.2	1.2	1,2	1,2	1,2	1,1
**Femoral hernia**	**Year**									
	**2000**	**2001**	**2002**	**2003**	**2004**	**2005**	**2006**	**2007**	**2008**	**2009**
***Total number***	536	510	513	462	516	465	439	471	620	553
rate x 100,000/year	11.9	11.2	11.3	10.1	11.1	9.9	9.3	9.9	12.8	11.3
***Ordinary admission (n.)***	359	307	317	261	291	236	233	227	216	211
Day of stay	1824	1368	1375	1166	1429	1015	1046	1057	1011	1057
Mean Length of stay	5.1	4.5	4.3	4.5	4.9	4.3	4.5	4.7	4.7	5.0
% Ordinary admission	67.0%	60.2%	61.8%	56.5%	56.4%	50.8%	53.1%	48.2%	34.8%	38.2%
% Urgent ordinary admission	54%	56%	59%	64%	73%	70%	73%	75%	77%	84%
***Day surgery (n.)***	177	203	196	201	225	229	206	244	188	150
n. visit	331	370	369	361	377	375	385	434	303	262
% Day surgery	33.0%	39.8%	38.2%	43.5%	43.6%	49.2%	46.9%	51.8%	30.3%	27.1%
***Ambulatory (n)***									216	192
% ambulatory									34.8%	34.7%
% Not ordinary admission	33.0%	39.8%	38.2%	43.5%	43.6%	49.2%	46.9%	51.8%	65.1%	61.8%
Mean admission days indicator	2.6	2.0	1.9	1.7	1.5	1.4	1,4	1,3	1,2	1,2

Concerning the type of healthcare service involved, there was a decrease in the proportion of procedures performed in ordinary admission for IH (p _forAAPC_ <0.05) – only 13% of IH cases were inpatients in 2009, and for FH (p_forAAPC_ < 0.05) – with 38% of cases in the last year of our analysis.

Coinciding with the reduction in the use of inpatient care, there was an increase in the number of admissions for day surgery. The choice of ambulatory type of service had been limited for both IH and FH in the first 8 years of the decade analyzed (i.e. up until the feasibility of performing these operations in outpatients had been formally recognized), but in the last two years considered this solution was adopted for 29% of IH repairs and 35% of FH repairs, thereby helping to reduce the hospitalization rates.

Finally, no cases of death were recorded for inpatients or outpatients undergoing hernia repair in our region, neither during their hospital stay nor on the days of procedures completed as outpatient care.

## Discussion

This study analyzed the epidemiological aspects of a decade of IH and FH repair surgery in the Veneto Region.

The figure found for IH repairs performed between 2000 and 2009, with an AIR of 291.2 procedures per 100,000 population per year, is consistent with the literature [20]. This figure tended to rise over the years, as seen in other countries [13].

There was a gradual decline in the use of conventional hospital stays, for IH repair (thanks to a careful selection of patients eligible for day surgery) and also for FH repair, albeit to a lesser degree (possibly because of the larger number of procedures in urgently admitted patients). Other international studies have seen a similar reduction in the use of inpatient procedures thanks to several factors, including surgeons’ greater use of local anesthesia for elective inguinal hernia repair [21], which has coincided with higher rates of surgery in day-hospital patients [22] and a less restrictive selection of cases eligible for day surgery. The selection criteria for patients amenable to day surgery were previously more restrictive, including only patients with a low risk of complications (ASA I-II, age limits, operating times <1 h, no severe obesity, etc.). Such strict selection criteria are becoming less common and day surgery for IH repair is now considered an option for all patients who have adequate home care [23,24]. Another reason supporting the transfer of patients to outpatient clinics or day surgery for unilateral IH and FH repair is the very low rate of complications, the most common of which is hernia recurrence. A large study conducted in Denmark identified a hospital readmission rate for hernia of 1.8% [25]. Our data indicate that no inpatient deaths occurred among patients operated over a ten-year period. Observational studies on different surgical techniques have shown that, although the “tension-free” approach under local anesthesia seems to be the most suitable procedure in outpatient surgery, other techniques can be equally effective. Only the wide, open, pre-peritoneal approach (the Stoppa technique) has reportedly not been used as a day surgery procedure. We nonetheless found, for both types of hernia, that hospitalization was longer for meshless procedures. These data are consistent with the finding reported in the Cochrane review on open-mesh versus meshless procedures for groin hernia repair [26] that the mesh groups generally had a shorter hospital stay and returned more quickly to their usual activities, probably because mesh repairs coincided with a more limited impairment of physical activity and less frequent persistent pain than non-mesh repairs [26].

In addition to clinical considerations, the appropriate use of health care resources (which includes avoiding recourse to hospitalization in all cases in which the diagnostic-therapeutic process does not require inpatient care) is also mandatory from the organizational point of view, to strike the right balance between supply and demand for services, on which the sustainability of the national health system depends [27]. The Health Ministry data in 2009 indicates that 78.7% of the related hospital admissions, in not complicated patients >17y for femoral and inguinal hernia repair in Veneto, involved day hospital services, whereas the national figure for this proportion in 2009 was 47.8% [28].

Our data also show that the number of days spent in hospital for ordinary admissions did not change over the decade considered. A likely explanation for this is that, despite gradual improvements with time in the health care provided during the patients’ stay, the greater selection of patients referred for ordinary hospital admissions over the years altered the case mix of patients involved. Moreover for the purposes of assessing the delivery of hospital care and thereby gauging whether hospital stays have changed over the years (whatever the type of health care service involved), we calculated the mean days of admission indicator, which dropped significantly over the course of our observation period (a decade). Although our data show that AIR and the number of interventions for FH remained broadly stable over the decade, unlike other studies, we found an increase in DS when this care model was implemented [13,20].

Our proxy efficiency indicator (corresponding to the length of hospital stays for scheduled surgery) can also be seen as a useful monitoring tool for programming purposes, for orienting appropriate hospital allocation decisions, given the dynamism with which day surgery and outpatient surgery are developing. These latter solutions can help to make hospitalizations more efficient, to ensure the sustainability of the NHS, and to offer candidates for scheduled surgery shorter waiting times. Concerning this last aspect, the average waiting time for inguinal or femoral hernia repair in Europe stood at around 12.4 weeks in 2001 [13], while in the UK the average waiting time was 15 weeks for unilateral inguinal and femoral hernias without gangrene in 2006 [20].

Further motivation to use day surgery or outpatient schemes could also come from the procedures for refunding medical expenses. In countries like the USA, day surgery procedures are reimbursed at the same rate as inpatient surgery, preventing any opportunistic behavior related to economic issues. In some countries, such as the UK, day surgery procedures are even financed at higher rates than inpatient procedures to prompt healthcare providers to become aligned with the national target to convert most surgical operations into day surgery procedures [13]. The Veneto Regional Authority has set a specific regional target of 13% (based on a review of the historical data for the region and of the international literature) for inpatient IH-FH treatment: when this target is exceeded, the price paid for ordinary admission for hernia repair drops from €1386 to €1040 to encourage a limitation of the use of inpatient care for IH-FH treatment [19].

## Conclusion

The Veneto Region’s policy is consistent with the Italian National Pact for Health 2010–2012, which explicitly calls for a reduction in the hospitalization rates, to be achieved by means of a number of actions including referring candidates for surgical procedures to day surgery and outpatient clinics instead of conventional hospitalization wherever possible in order to make the best possible use of the hospital beds and incur the lowest possible costs for the Italian national health service [29].

## Abbreviations

IH: Inguinal hernia; FH: Femoral hernia; AIR: Annual intervention rate; DS: Day surgery.

## Competing interests

The authors have no potential conflicts of interest to disclose.

## Authors’ contributions

MS: conceptualized the study, carried out the statistical analyses and approved the final manuscript as submitted. DM: data interpretation, reviewed and revised the manuscript, and approved the final manuscript as submitted. AB: designed the study, carried out the statistical analyses, reviewed and revised the manuscript, and approved the final manuscript as submitted. GC: conceptualized the study, and approved the final manuscript as submitted. CZ: wrote the initial manuscript and approved the final manuscript as submitted. CB and TB critically reviewed the manuscript, and approved the final manuscript as submitted. VB: conceived the study, and participated in its design and coordination, reviewed and revised the manuscript, and approved the final manuscript as submitted. All authors read and approved the final manuscript.

## Pre-publication history

The pre-publication history for this paper can be accessed here:

http://www.biomedcentral.com/1472-6963/13/349/prepub
